# Supplementary material for: In-Line Aerosol Therapy via Nasal Cannula during Adult and Paediatric Normal, Obstructive, and Restrictive Breathing
Source: Pharmaceutics. 2023 Nov 27;15(12):2679. doi: 10.3390/pharmaceutics15122679 (PMC10747070; doi:10.3390/pharmaceutics15122679)
Supplement: Supplementary file 1 [file pharmaceutics-15-02679-s001.zip › pharmaceutics-2713399-supplementary.pdf]

Supplementary Materials

Table S1. The effects of supplemental gas flow rate (LPM) and lung health on the tracheal dose (%) available in spontaneously breathing adult receiving in-line aerosol therapy with HFNO. P-values < 0.05 considered significant.

Flow Rate (LPM)	Lung Health	Tracheal dose (%) (mean ± SD)	P - value
6 (Low flow mode)	Healthy	16.58 ± 0.36	0.000
	Restrictive	15.12 ± 0.54	
	Obstructive	7.70 ± 0.51	
10	Healthy	15.99 ± 0.71	0.000
	Restrictive	15.63 ± 0.30	
	Obstructive	8.42 ± 0.70	
30	Healthy	0.73 ± 0.13	0.012
	Restrictive	1.20 ± 0.23	
	Obstructive	0.90 ± 0.25	
50	Healthy	0.38 ± 0.09	0.005
	Restrictive	0.23 ± 0.06	
	Obstructive	0.50 ± 0.15	

Table S2. The effects of supplemental gas flow rate (LPM) and lung health on the tracheal dose (%) available in spontaneously breathing paediatric model receiving in-line aerosol therapy with HFNO. P-values < 0.05 considered significant.

Flow Rate (LPM)	Lung Health	Tracheal dose (%) (mean ± SD)	P - value
3	Healthy	2.93 ± 0.20	0.000
	Restrictive	0.91 ± 0.20	
	Obstructive	4.07 ± 0.45	
7	Healthy	0.71 ± 0.15	0.030
	Restrictive	0.52 ± 0.26	
	Obstructive	0.88 ± 0.12	
